# Differential Effects of MicroRNAs on Glioblastoma Growth and Migration

**DOI:** 10.3390/genes4010046

**Published:** 2013-03-04

**Authors:** Duane Jeansonne, Marco Pacifici, Adam Lassak, Krzysztof Reiss, Giuseppe Russo, Jovanny Zabaleta, Francesca Peruzzi

**Affiliations:** 1 LSU Health Sciences Center, Medical School, Stanley S. Scott Cancer Center, 533 Bolivar Street, New Orleans, LA 70112, USA; E-Mails: djean1@lsuhsc.edu (D.J.); mpacif@lsuhsc.edu (M.P.); alassa@lsuhsc.edu (A.L.); kreiss@lsuhsc.edu (K.R.); jzabal@lsuhsc.edu (J.Z.); 2 Sbarro Institue for Cancer Research and Molecular Medicine, Temple University, Philadelphia, PA, USA; E-Mail: grusso@temple.edu

**Keywords:** Glioblastoma, microRNA, mTOR, CYR61

## Abstract

Glioblastoma multiforme is characterized by rapid proliferation, aggressive metastatic potential, and resistance to radio- and chemotherapy. The matricellular protein CYR61 regulates cellular proliferation and migration and is highly expressed in Glioblastomas. MicroRNAs are 22-nucleotides long RNAs that regulate gene expression post-transcriptionally. Here, we utilized the LN229 glioblastoma cell line and found that CYR61 is a target of miR-136, miR-155, and miR-634. Over-expression of miR-136 and miR-634 miRNAs negatively affected proliferation, but not migration, while expression of miR-155 reduced migration but did not affect the proliferation of LN229 cells. Investigation of the molecular mechanisms affected by expression of miR-634 revealed an increased phosphorylation of p70S6 kinase, suggesting an induction of the mammalian target of rapamycin (mTOR) complex 1 pathway. Additionally, in miR-634 overexpressing cells, TSC2, a negative regulator of mTOR signaling, was found to be decreased. Altogether, our study provides insights on the differential roles of miRs-136, -155, and -634 in regulating glioblastoma cell growth and migration, and how microRNAs could be manipulated to decrease the aggressiveness and metastatic potential of tumor cells.

## 1. Introduction

Glioblastoma multiforme (GBM) is a devastating malignancy with a median survival of approximately 15 months [1]. GBM develops as a result of additive genetic mutations leading to malignant transformation. In Glioblastomas, these genetic changes and aberrant gene regulation involve many well-known genes and pathways, including IGF-1, RAS, p53, PTEN, AKT, among others [2,3,4,5,6,7]. 

Cysteine-rich 61 (CYR61) is a member of the CCN (CYR61, CTGF, and Nov) family of matricellular proteins [8]. The CCN family of proteins consists of six members: CCN1 (CYR61), CCN2 (connective tissue growth factor, CTGF), CCN3 (nephroblastoma-overexpressed, Nov), CCN4 (Wnt-inducible secreted protein-1, WISP-1), CCN5 (WISP-2), and CCN6 (WISP-3) [9]. Members of the CCN family each contain a secretory signal sequence and are found to associate with the extracellular matrix (ECM). CYR61 is a multidomain, cysteine-rich, heparin-binding protein that mediates the binding of multiple protein complexes and interacts with cellular integrins [9]. CYR61 regulates many cellular functions, including proliferation, migration, angiogenesis, differentiation, adhesion, survival, and apoptosis [10,11,12,13]. It has been well established that aberrant CYR61 expression and signaling can lead to tumorigenesis [14,15,16,17,18]. In fact, the expression of CYR61 has been found to be elevated in many cancers including, breast, colon, pancreatic, melanomas, and gliomas [9,14,19].

The mTOR signaling pathway controls numerous aspects of cellular biology, including proliferation, cell cycle progression, translation, and cell size. mTOR is a serine/threonine kinase that can be found in two distinct complexes, mTORC1 and mTORC2. The complex mTORC1 can be inhibited by rapamycin and is responsible for controlling protein synthesis and the phosphorylation of downstream targets such as p70S6 kinase [20]. Signaling through mTORC1 is dependent on the activity of AKT and ERK1/2. These kinases phosphorylate and inhibit the activity of Tuberin/TSC2, a protein that forms a complex with Hamartin/TSC1 to act as a principle negative regulator of mTORC1 [21]. Although less is known about mTORC2, this complex has been shown to be rapamycin-insensitive and is known to play roles in cell survival, metabolism and cytoskeletal organization [22].

MicroRNAs (miRNAs) are 19-25 nucleotide species of non-translated RNA that bind to the 3’UTR of target mRNAs with incomplete complementarity [23]. The binding of a miRNA to the 3’UTR of a gene results in translation inhibition or degradation of the mRNA transcript. In this study, we investigated the role of miRNAs in regulating CYR61 expression, which we also found to be down-regulated by treatment of the cells with fenofibrate, a hypolipidemic drug with anti-cancer properties. Our work demonstrated that fenofibrate treatment, miR-136, miR-155, and miR-634, target CYR61. Overexpression of miR-136 and miR-634 negatively affected cell proliferation with no impairment of migration, whereas miR-155 was found to impair the migration of LN229 cells. Surprisingly, although addition of CYR61 enhanced migration of control cells, it did not restore migration potentials of cells expressing miR-155. Investigation of the molecular mechanisms controlled by these microRNAs revealed an induction of the mTORC1 signaling pathway, which is likely involved in maintaining the proliferation and migration potentials of miR-expressing cells, despite the down-regulation of CYR61. 

## 2. Results and Discussion

### 2.1. CYR61 Is a Target for miR-136, miR-155, and miR-634

**Figure 1:**
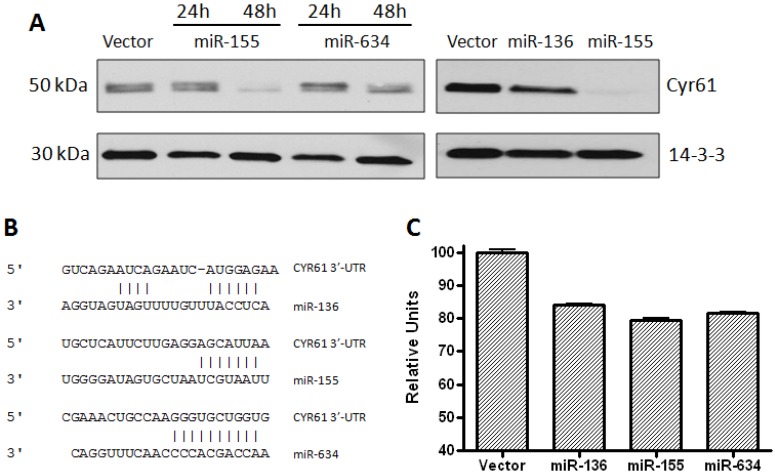
CYR61 is a target for miR-136, miR-155, and miR-634. **(A)** Western blot showing levels of CYR61 in LN229 cells transfected with miRNA-expressing Block-iT plasmids. 14-3-3 antibody was used to show equal loading of cellular lysates. **(B)** Diagram depicting the predicted binding sites of the microRNAs to the 3’UTR of *cyr61* mRNA sequence. **(C)** Luciferase assays of HeLa cells co-nucleofected with *cyr61* 3’UTR and miRNA-expressing plasmids. Firefly and *Renilla* values were determined at 24 h post-nucleofection of cells. The data represents the ratio between *Renilla* values and firefly internal control for each group. Assays were performed in duplicate.

**Figure S1:**
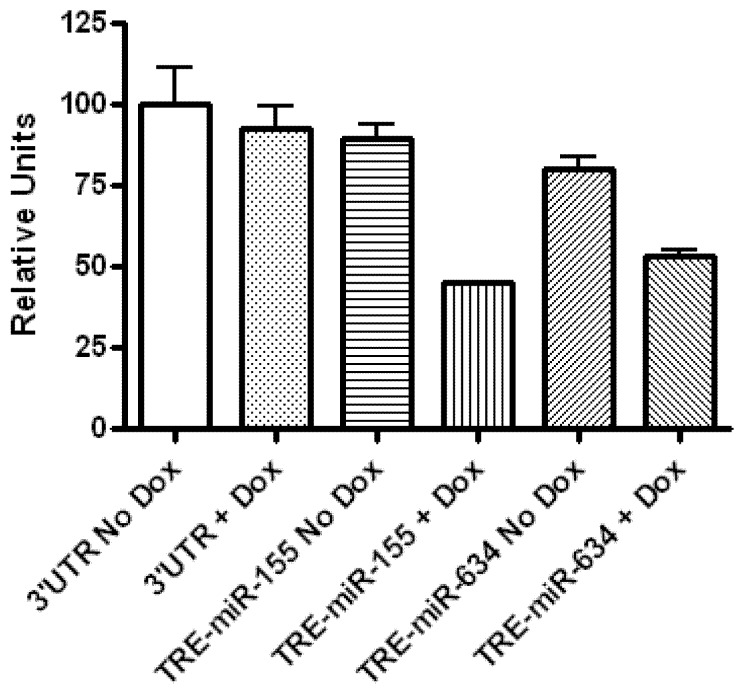
Inhibitory effect of induced miR-155 and miR-634 expression on *cyr61*. Luciferase assay of LN229 cells transiently co-transfected with the *cyr61* 3’UTR and empty vector or with TRE3G-IRES-miRNA expressing plasmids. miRNA-expressing plasmids were under the control of a doxycycline-inducible vector. Firefly and *Renilla.* values were determined at 24 h post-transfection. The data represent the ratio between *Renilla.* values and firefly internal control for each group (n = 2).

Recently, miR-155 was reported to block the migration of HTR-8/SVneo cells by directly targeting the 3’UTR of CYR61 and down-regulating its expression [24]. Two additional miRNAs that were predicted, but never validated, to target the 3’UTR of CYR61 are miR-136 and miR-634. These microRNAs were selected for analysis in this study as they both have been reported to possess tumor suppressor activity. MiR-136 was shown to augment the apoptotic effects of chemotherapy against glioma cells [25] and miR-634 demonstrated anti-proliferative effects against prostate cancer cells [26]. To confirm that CYR61 is a target for miRs-136, -155, and -634, we evaluated the expression of CYR61 protein following transient overexpression of the miRNAs. Expression levels of the microRNAs were determined by qRT-PCR before and after transient transfection (data not shown). Expression of CYR61 protein was determined in LN229 cells at 24 h and 48 h after transfection with miRNAs. Overexpression of each individual miRNA resulted in down-regulation of CYR61 with the strongest down-regulation at 48 h (Figure 1A). As expected, transfection of LN229 cells with empty vector did not result in any decrease in the level of CYR61 expression. Of note, the degree of CYR61 protein down-regulation upon expression of each microRNA is different, with miR-155 being the most effective, followed by miR-634 and then miR-136. To determine whether these miRNAs could block the expression of CYR61, a functional assay using the whole 3’UTR of this target was performed. The genomic sequence corresponding to the 3’UTR of CYR61 was cloned into the psiCHECK2 vector, which contains *Renilla* and firefly luciferase reporter sequences. The CYR61 3’UTR sequence contains one putative binding site for each microRNA (miR-136, miR-155, and miR-634) as predicted by TargetScan microRNA gene target prediction database (Figure 1B). Luciferase assays were performed by nucleofecting HeLa cells with the psiCHECK2 vector containing the 3’UTR along with miR-expressing or empty Block-iT plasmids. At 24 h post-transfection, luciferase assays were performed and results are shown in Figure 1C. The ratio of *Renilla*/firefly luciferase for each microRNA was normalized to empty plasmid control (100%). A 20% decrease in the *Renilla*/firefly luciferase ratios was observed when the psiCHECK vector was co-transfected with miRNA-expressing plasmids, as compared to empty plasmid control. This result was repeated in LN229 cells with miR-155 and miR-634 under the control of a doxycycline-inducible vector system. Here, a 50% reduction in the *Renilla*/firefly ratio was observed upon induction of microRNA expression by doxycycline (Figure S1). Taken together, these results confirm that miR-136, miR-155, and miR-634 directly target the 3’UTR of CYR61.

### 2.2. Glioblastoma Growth and Migration are Differentially Affected by miR-136, miR-155, and miR-634

**Figure 2:**
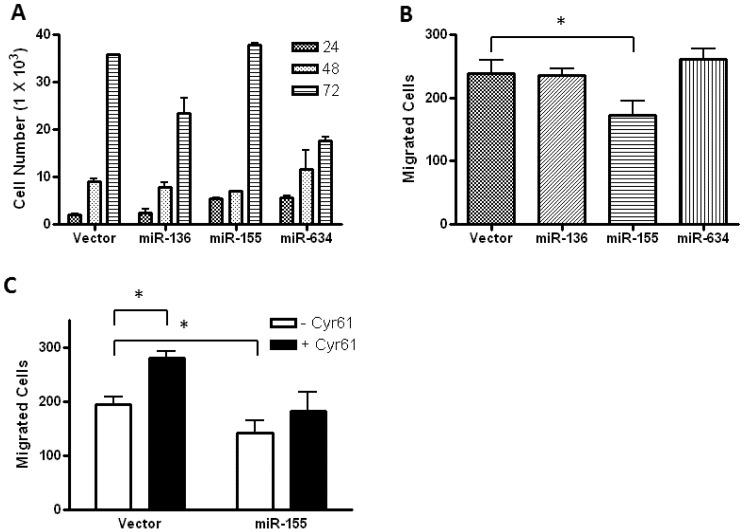
Differential effects of miR-136, miR-155, and miR-634 on cell growth and migration of LN229 cells. **(A)** Plot showing the growth of miRNA-expressing LN229 cells at 24 h, 48 h, and 72 h after plating. Experiment was repeated twice, each in duplicate. **(B)** Migration assay of miRNA-expressing LN229 cells. At 48 h post-transfection, GFP^+^ cells were sorted onto 8 µm filter membranes and allowed to migrate for 16h (see Experimental Section). Experiment was repeated twice, each in triplicate. **(C)** Migration assay in the presence of recombinant CYR61. LN229 cells were nucleofected with plasmid expressing miR-155 or empty vector and plated onto membranes with or without the presence of 200 ng/mL CYR61. Migration assays were performed as described above. * indicates values that are statistically significantly different (p ≤ 0.05).

To get insights into the biological outcome of the selected microRNAs, we determined the effect of miRNA overexpression on the growth of LN229 cells. To this end, we took advantage of the Block-iT microRNA expression vector that allows visualizing positively transfected cells via expression of a green fluorescent protein (GFP). Cells were transiently transfected, sorted for GFP expression into 12-well plates, and counted at 24 h, 48 h, and 72 h. Cell growth is indicated as the number of viable cells counted by flow cytometry at each time point using the Guava ViaCount assay. Figure 2A shows a steady increase in the number of viable cells for each group up to 72 h. The largest increases in cell growth occur between the 48 h and 72 h time points. Compared to vector alone, the growth of LN229 cells overexpressing miR-136 and miR-634 was reduced by about 30% and 50%, respectively. Analysis of DNA content in LN229 cells expressing these microRNAs did not reveal any significant changes in cell cycle distribution, as compared to vector alone (data not shown). MiR-155 overexpression had no effect on cell growth in this experiment. 

**Figure S2:**
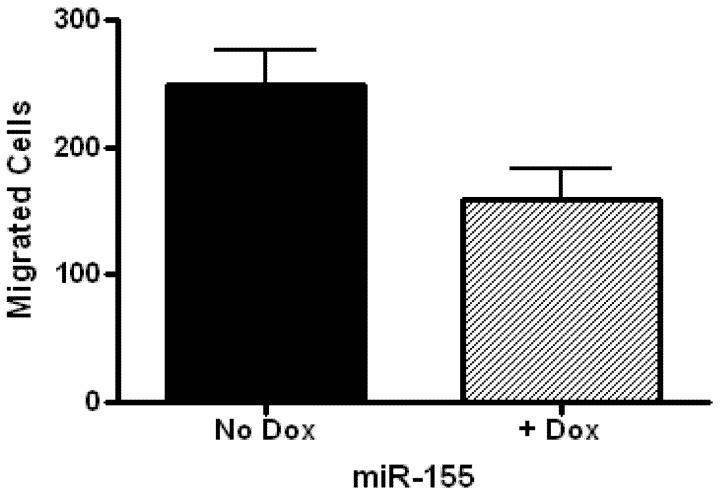
Migration assay of LN229 cells expressing miR-155 under the control of a doxycycline-inducible vector. Expression of miR-155 was induced for 48 h before plating onto 8 µm membranes, as detailed in Experimental Section.

In order to examine the effect of these miRNAs on the invasive potential of Glioblastoma, we conducted cell migration assays with transiently transfected LN229 cells. Overexpression of miRs-136 and -634 did not significantly affect cell migration, as compared to that of cells transfected with vector alone. However, overexpression of miR-155 did significantly reduce cell migration across the membrane (Figure 2B). This result was confirmed using stably-transfected LN229 cells expressing miR-155 under the control of a doxycycline-inducible promoter (Figure S2). Since CYR61 is secreted by cancer cells and has been shown to promote their migration [7,24], we reasoned that addition of soluble CYR61 to miR-155-transfected cells should restore the migratory capability of these cells. The migration assay was repeated as in Figure 2B in the presence of 200 ng/mL recombinant CYR61 protein. Shown in Figure 2C an increase in migration of cells expressing vector alone can be seen when CYR61 is added to the medium. However, CYR61 did not increase the migration of cells expressing miR-155 in a statistically significant manner. Although this experiment demonstrates that CYR61 increases the migratory capacity of LN229, it suggests that other miR-155 targets in addition to CYR61 may be critical for the migration of LN229 cells.

Despite a dramatic reduction in CYR61 expression, miR-155 transfected cells do not show deficits in proliferation, suggesting that CYR61 is not strongly involved in controlling cell growth. This argument is reinforced by the fact that cells expressing miR-136 and miR-634, in which CYR61 is still highly detectable, grow less efficiently than the miR-155 transfected cells.

### 2.3. Expression of miRs-136, -155, and -634 Enhance ERK, AKT, and p70S6K Signaling Pathways

**Figure 3:**
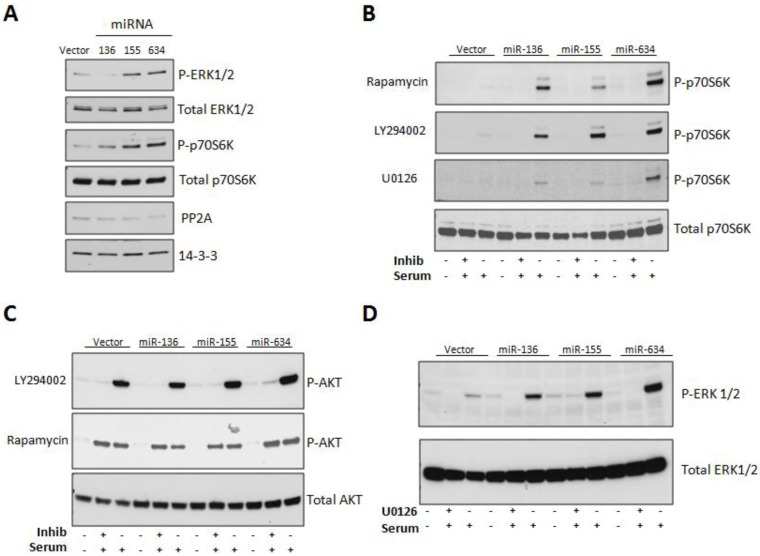
Expression of microRNAs (-136, -155, or -634) up-regulate mTOR signaling in an AKT- and ERK1/2-dependent manner. **(A)** Western blot analysis of cells nucleofected with miRNA-expressing plasmids. Western blot of phospho- and total proteins was performed on lysates from LN229 cells that were transiently nucleofected with empty or miRNA-expressing plasmids for 48 h. **(B)** Western blot of phospho-p70SK (T389) and total p70SK. Western blot was performed on lysates from LN229 cells that stably express empty or miRNA-expressing plasmids and were pretreated with specific inhibitors (50 µM LY294002, 10 nM Rapamycin, or 15 µM U0126) for 1 h followed by stimulation with 10% serum for 1 h. **(C)** Western blot of phospho- and total AKT (S473). Cells were treated as described in panel B. **(D)** Western blot of phospho- and total ERK1/2 (T202/Y204). Cells were treated as described in panel B.

Since the rate of cell growth was reduced in miR-136 and miR-634 transfected cells, we next investigated which mitogenic signaling pathways are affected by expression of these miRNAs. Furthermore, the ERK, AKT, and p70S6K signaling pathways have been shown to be activated by CYR61 in a variety of cell types [7,27,28,29]. As our selected miRNAs inhibit CYR61 expression, we expected an attenuation of these pathways in our cellular model. Therefore, we examined the activation of AKT, ERK1/2, and p70S6 kinase in response to miRNA overexpression in LN229 cells. As these experiments require high efficiency of transfection, we switched from standard Lipofectamine methods to nucleofection, therefore avoiding the sorting step. The Western blot in Figure 3A shows that all three miRNAs significantly increased the phosphorylation of ERK1/2 (T202/Y204), and p70S6 kinase (T389), as compared to empty vector control. As PP2A is known to dephosphorylate ERK1/2, we reasoned that its expression could be impaired in the miR-expressing cells, in which phosphorylation of ERKs is increased. However, no significant changes were observed in the levels of this protein phosphatase (Figure 3A, fifth panel). The mTOR pathway has been shown to promote cell growth or senescence, depending on the cellular context [20,30,31]. Therefore, we next sought to determine the contribution of PI3-kinase, ERK1/2, and mTOR Complex 1 (mTORC1) signaling to the activation of p70S6 kinase, which is phosphorylated in response to mTOR pathway activation. LN229 cells stably expressing our miRNAs of interest were serum-starved for 24 h, pretreated with PI3-kinase inhibitor LY294002 (50 µM), MEK1 inhibitor U0126 (15 µM), or mTORC1 inhibitor rapamycin (10 nM) for 1 h, followed by 1 h treatment with 10% FBS. Figure 3B shows that the phosphorylation of p70S6 kinase (T389) was dependent on all three inhibitors. Lysates of cells collected at Time zero (T_0_), which were not stimulated with 10% FBS, show no significant phosphorylation of p70S6 kinase. Importantly, densitometric analysis revealed that the stable miR-136, -155, and -634 expressing cell lines showed 3.6 (+/− 1.3), 3.7 (+/− 0.1), and 2.6 (+/− 0.4) respective fold increases in p70S6 kinase phosphorylation as compared to vector control. This result is in agreement with that found under conditions of transient overexpression of the miRNAs (Figure 3A).

A Western blot for phospho-AKT (S473) was conducted using lysates incubated with LY294002 and rapamycin (Figure 3C). As expected, LY294002 blocked AKT phosphorylation at S473 (Figure 3C, top panel) and rapamycin had no effect on AKT phosphorylation (Figure 3C, middle panel). MiRNA expression did not result in a significant increase of AKT phosphorylation, as compared to vector control. However, densitometric analysis of Western blots from stable miR-136, -155, and -634 expressing cells did show 2.2 (+/− 0.6), 3.3 (+/− 0.01), and 5.5 (+/− 0.4) respective fold increases in ERK1/2 (T202/Y204) phosphorylation as compared to vector-expressing cells. This phosphorylation was efficiently blocked by the MEK1 inhibitor U0126 (Figure 3D).

**Figure 4:**
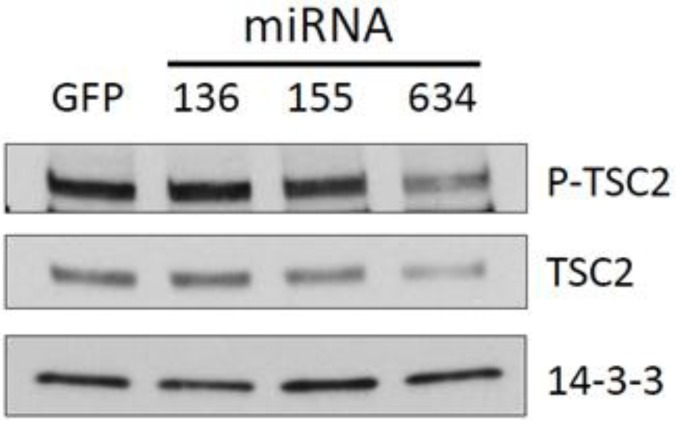
miR-634 reduces the expression of TSC2, a negative regulator of mTOR signaling. Western blot of phospho- and total TSC2. Western blot analysis was performed on lysates from cells that were nucleofected with empty or miRNA-expressing plasmids for 48 h. 14-3-3 antibody was used to show equal loading of cellular lysates.

In addition to targeting the 3’UTR of CYR61, we sought to determine whether down-regulation of this protein by the three selected miRNAs was dependent on the activation of ERKs, AKT, or mTOR pathways. Results indicated no significant changes in CYR61 levels upon inhibition of any of these kinases (data not shown). The protein TSC2 negatively regulates the mTOR pathway by binding and blocking the GTPase activity of Rheb, a positive regulator of mTORC1 activity [32]. Previous reports have shown that AKT and ERK1/2 can phosphorylate TSC2, resulting in its degradation or inactivation [33,34,35]. Therefore, we decided to investigate whether this mechanism of mTOR activation is occurring in LN229 cells overexpressing the miRs in our study. We performed a Western blot for total TSC2 and phospho-TSC2 at position S664, which is phosphorylated by ERK1/2, in cells overexpressing our selected miRNAs (Figure 4). The results show that, among the three microRNAs, only overexpression of miR-634 resulted in a decreased amount of phosphorylated (S664) and total TSC2 in LN229 cells. Overexpression of miR-136 or miR-155 did not appear to significantly decrease total or phospho-TSC2.

### 2.4. Fenofibrate Down-Regulates the Expression of CYR61 in LN229 Glioblastoma Cells

Fenofibrate is a member of a class of drugs known to stimulate the activity of the transcription factor, peroxisome proliferator activated receptor α (PPARα). This drug is used clinically to lower levels of cholesterol, triglycerides, low-density lipoprotein, and very low-density lipoprotein levels in patients. However, fenofibrate also possesses antitumor activities, which have been demonstrated in multiple cancers, including glioma, medulloblastoma, endometrial, and melanoma, among others [3,36,37,38].

**Figure 5:**
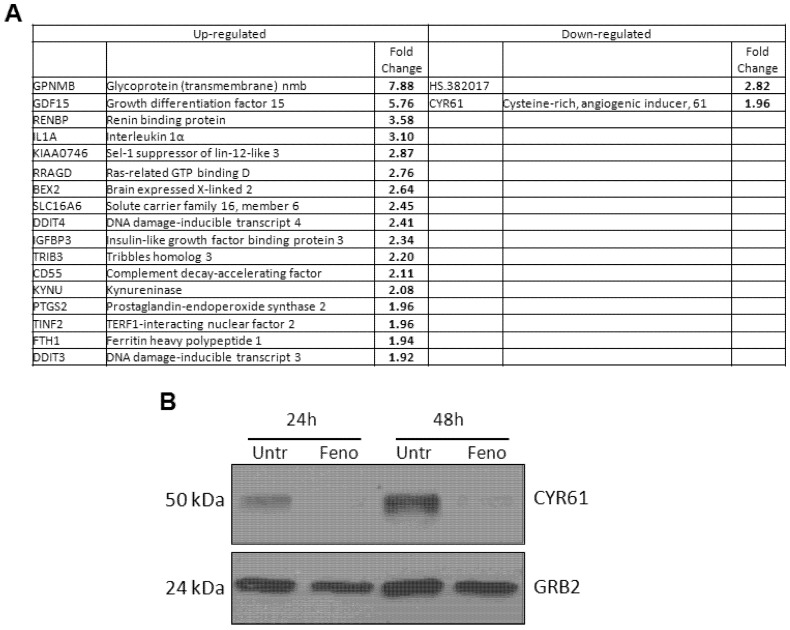
**(A)** List of fenofibrate regulated mRNAs in LN229 cells. Fold change values reflect mRNA levels in cells treated with 50 µM fenofibrate *versus* untreated cells. Results shown are the average of two independent experiments. **(B)** Down-regulation of CYR61 protein expression in LN229 glioblastoma cells after fenofibrate treatment. Western blot of CYR61 was performed on lysates from cells that were untreated or treated with fenofibrate (50 µM) for 24 h or 48 h. GRB2 antibody was used to show equal loading of cellular lysates.

The effect of fenofibrate treatment on gene expression in LN229 cells was evaluated by mRNA microarray analysis. Figure 5A shows the results from this analysis where several transcripts were differentially regulated in a statistically significant manner following fenofibrate treatment. Bioinformatics analysis suggested a deregulation of genes involved in proliferation and migration. Of the down-regulated transcripts, only one contained the sequence for a protein-coding gene, CYR61. The down-regulation of the CYR61 transcript observed in the microarray was further validated by Western blot analysis on protein extracts derived from fenofibrate-treated cells and controls. Results in Figure 5B show a dramatic down-regulation of CYR61 protein upon fenofibrate treatment for 24 and 48 hrs. As fenofibrate blocks the growth of LN229 cells [39] and reduces the levels of CYR61, we further tested whether the addition of CYR61 may reverse the cytotoxic effects of this inhibitor. However, LN229 cells treated with fenofibrate in the presence of soluble recombinant CYR61 did not demonstrate increased survival over cells treated with fenofibrate alone (data not shown).

Since CYR61 3’UTR is predicted to be a target of miR-136, miR-155, and miR-634, we next sought to investigate the role of our selected microRNAs in the fenofibrate-mediated down-regulation of CYR61. Expression levels of CYR61-targeting miRNAs (136, 155, and 634) in LN229 cells treated with fenofibrate were measured by quantitative real-time PCR (qRT-PCR) as described in the Experimental Section. However, no statistically significant changes were observed at 24 h and 48 h post-treatment (data not shown).

### 2.5. Expression of miR-634 Partially Protects Cells From Fenofibrate-Treatment in a Clonogenic Assay

**Figure 6:**
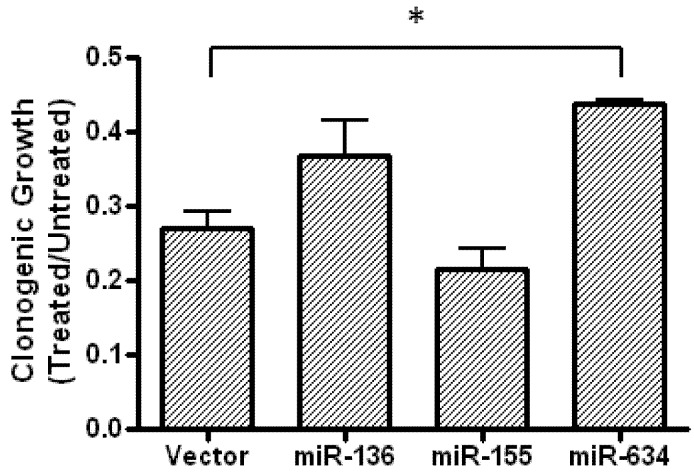
LN229 cells expressing miR-634 are less sensitive to the growth suppressive effects of fenofibrate. Clonogenic assay of LN229 cells stably expressing the indicated microRNAs. Experiment was repeated twice, each in duplicate. * indicates values that are statistically significantly different (p ≤ 0.05).

Although changes in the expression of our selected miRNAs were not detected in fenofibrate treated cells, we still reasoned that they could enhance the fenofibrate inhibitory effect on CYR61, perhaps sensitizing cells to fenofibrate treatment. Experiments were carried out to determine if clonogenic growth in the presence of fenofibrate is affected by miRNA expression. Cells stably expressing the indicated miRNAs were plated at very low density in 35 mm dishes (see Experimental section). After 24 h, fresh medium or medium containing 50 µM fenofibrate was added to the plates, and replaced every three days thereafter. After 14 days, cells were fixed and stained with crystal violet. The clonogenic density in the presence of fenofibrate was then normalized to clonogenic density in the absence of fenofibrate for each miRNA (Figure 6). The results show that, compared to cells expressing vector alone, the clonogenic density of miR-634-expressing cells was increased by about 50%. This result was also found when the experiment was repeated using LN229 cells with transient overexpression of miR-634 (data not shown).

## 3. Experimental Section

### 3.1. Cell Culture, Transfection, and Reagents

LN229 and HeLa cells were obtained from the American Type Culture Collection (Manassas, VA, USA). Cells were grown in Dulbecco’s Modified Eagle Medium (DMEM) supplemented with 10% fetal bovine serum, 100 units/mL penicillin, and 100 mg/mL streptomycin (Life Technologies, Rockville, MD, USA) and incubated at 37°C in 5% CO_2_. Fenofibrate was obtained from Sigma (St. Louis, MO, USA). Rapamycin was purchased from EMD Millipore (Darmstadt, Germany). LY294002 and U0126 were from Enzo Life Sciences (Farmingdale, NY, USA). Recombinant CYR61 protein was purchased from Abnova (Taipei, Taiwan).

For experiments requiring transient expression of miRNAs, 0.5 × 10^6^ cells were resuspended in 100 µL of Nucleofector Solution T, mixed with 2 µg of plasmid DNA, and nucleofected using Nucleofector Program X-01 (Lonza, Basel, Switzerland). For the experiments shown in Figure 3A, Figure 4A, and 4B, cells were seeded at a density of 4 × 10^5^ cells/60 mm dish and transfected using Lipofectamine 2000 (Life Technologies) according to the manufacturer’s instructions. Transfection using Lipofectamine 2000 was followed by cell sorting for enrichment of GFP^+^ cells. Cell sorting was performed using a FACSAria (BD Biosciences, San Jose, CA, USA). In some experiments, cells were washed twice with PBS and serum starved overnight in DMEM. After 24 h, fresh serum-free DMEM with or without the presence of inhibitors was added to plates. After 1 h, cells were stimulated with 10% FBS with or without the presence of inhibitors for 1 h at which time cells were collected by scraping.

### 3.2. Western Blot Analysis

LN229 cells were collected by scraping the plates in the presence of PBS, followed by centrifugation and disruption of the cell pellet in the appropriate volume of lysis buffer (50 mM HEPES, pH 7.5, 150 mM NaCl, 1.5 mM MgCl_2_, 1 mM EGTA, pH 8.4, 10% glycerol, 1% Triton X-100, 1 mM PMSF, 1 mM sodium orthovanadate, phosphatase inhibitor and protease inhibitor cocktails (Sigma)). Whole-cell lysates (30 to 70 µg) were separated on a 4%–15% SDS-PAGE gel (Bio-Rad, Hercules, CA, USA). CYR61 antibody (MAB4055) was purchased from R&D Systems (Minneapolis, MN, USA). Phospho-p70S6 kinase (T389) (#9234), total p70S6 kinase (#2708), phospho-ERK1/2 (T202/Y204) (#9106), total ERK1/2 (#9102), phospho-AKT (S473) (#4060), total AKT (9272) and TSC2 (#4308) antibodies were purchased from Cell Signaling Technology (Beverly, MA, USA). Phospho-TSC2 (S664) antibody (ab133465) was obtained from Abcam (Cambridge, MA, USA). Monoclonal antibodies specific to PP2A catalytic α subunit (610555) and GRB2 (610112) were from BD Transduction Laboratories (San Jose, CA, USA). 14-3-3 antibody (sc-1019) was obtained from Santa Cruz Biotechnology (Santa Cruz, CA, USA).

### 3.3. mRNA Microarray Analysis

LN229 Glioblastoma cells were treated with 50 µM fenofibrate for 24 h and 48 h. RNA was isolated from the cells at the indicated time points and subjected to nanodrop analysis to verify the quality of the RNA. mRNA microarray analysis was performed using a HumanWG-6 Expression BeadChip (Illumina, San Diego, CA) covering over 37,000 genes, including control gene sequences. Bioinformatics analysis for miR predicted gene targets was performed using GenGo software and miRWalk database (http://www.umm.uni-heidelberg.de/apps/zmf/mirwalk/).

### 3.4. Quantitative RT-PCR

RNA was isolated from cells using the miRVana miRNA extraction kit (Ambion, Austin, TX, USA). RNA was reverse transcribed to cDNA using the High Capacity cDNA Reverse Transcription kit containing TaqMan® primers specific for each respective miRNA (Applied Biosystems, Carlsbad, CA, USA). Quantitative real-time PCR for detection of mature miRNAs (miR-136, miR-155, and miR-634) or control U6 was performed using TaqMan® RT-PCR primers (Applied Biosystems). For each sample containing cDNA template, RT-PCR was performed in duplicate using a LightCycler 480 Real-Time PCR System from Roche (Indianapolis, IN, USA). Each sample was normalized using U6 control and relative quantification of miRNA expression was calculated using the comparative Ct (ΔΔCt) method, as described in our previous publications [40,41,42].

### 3.5. Cloning for miRNA Functional Analysis

The mature hsa-miR-136 sequence used corresponds to miRBase acession #: MIMAT0000448. The following oligos were used to clone hsa-miR-136 into the Block-iT Pol II miR RNAi Expression Vector (Invitrogen): forward, 5’-TGCTGACTCCATTTGTTTTGATGATGGAGTTTTGGCCACT-GACTGACTCCATCATAAACAAATGGA and reverse, 5’-CCTGTCCATTTGTTTATGATGG-AGTCAGTCAGTGGCCAAAACTCCATCATCAAAACAAATGGAGTC. For hsa-miR-155, the mature sequence (miRBase accession #: MIMAT0000646) was cloned using the oligos: forward, 5’-TGCTGTTAATGCTAATCGTGATAGGGGTGTTTTGGCCACTGACTGACACCCCTATCGATTAGCATT and reverse, 5’-CCTGAATGCTAATCGATAGGGGTGTCAGTCAGTGGCCAAAACA-CCCCTATCACGATTAGCATTAAC. For the cloning of mature hsa-miR-634 (miRBase accession #: MIMAT0003304), the oligos used were: forward, 5’-TGCTGAACCAGCACCCCAACTTT-GGACGTTTTGGCCACTGACTGACGTCCAAAGGGGGTGCTGGT and reverse, 5’-CCTGA-CCAGCACCCCCTTTGGACGTCAGTCAGTGGCCAAAACGTCCAAAGTTGGGGTGCTGGTTC. All oligos were cloned into the Block-iT Pol II miR RNAi Vector (Invitrogen) according to the manufacturer’s protocol.

The genomic sequence corresponding to the 3’UTR of CYR61 was PCR amplified from LN229 cells. This PCR product was ligated into the multiple cloning site downstream of the *Renilla* luciferase reporter gene in the PsiCHECK2 vector (Promega, Madison, WI). This vector also contains a firefly luciferase reporter sequence, which allows for normalization of transfection efficiency. The primers used were: forward, 5’-CTACTCGAGTTTCCAGGGCACACCTAGAC, and reverse, 5’-CTACTCGAGGCTTAATTCACTGCTGTATCC, containing the XhoI restriction site (underlined). 

### 3.6. Dual Luciferase Assay

HeLa cells were nucleofected with 2 µg of plasmid DNA according to the manufacturer’s protocol (Lonza, Basel, Switzerland). Nucleofected cells were plated at a density of 8 × 10^4^ cells/well in a 12-well plate. After 24 h, cells were harvested and lysates were assayed for luciferase activity with the Dual-Luciferase Reporter Assay System (Promega) using a Synergy 2 microplate reader (BioTek Instruments, Inc., Winooski, VT, USA). Relative units of *Renilla* luciferase activity were normalized to firefly luciferase internal control for each sample. Experiments were performed in duplicate.

MicroRNA target validation was also carried out in LN229 cells with miRNA expression under control of the Tet-on inducible vector system (Clontech). LN229 cells were plated at a concentration of 8 × 10^4^ cells/well in a 12-well plate. The next day, cells were transfected with 0.6 µg of DNA/well using Lipofectamine reagent (Life Technologies) according to manufacturer’s protocol. pcDNA3 was used to maintain a constant amount of DNA in each transfection. After 24 h, cells were harvested and assays were performed as described above.

### 3.7. Cell Growth Assay

LN229 cells were transiently transfected with empty or miR-expressing Block-iT plasmids and sorted into 12-well plates at a density of 10,000 cells/well. At 24 h, 48 h, and 72 h after plating, cells were trypsinized and resuspended in 200 µL of PBS. Cells were then diluted 10-fold into ViaCount reagent (Guava Technologies, Hayward, CA) and viable cells were counted by flow cytometry using the Guava easyCyte 8HT instrument (Guava Technologies).

### 3.8. Cell Migration Assay

Empty plasmid or microRNA-expressing LN229 cells were plated at a density of 25,000 cells into wells in a 24-well plate containing 8 µm filter membranes (BD Biosciences). Cells were allowed to migrate for 16h. Next, the top side of the filter membranes were wiped free of cells and washed with PBS. Cells were fixed, stained with crystal violet, and counted under light microscopy. The experiment was performed in triplicate.

### 3.9. Clonogenic Growth Assay

Untransfected or miR-expressing LN229 cells were plated at a density of 1000 cells/35-mm dish. After 24 hrs, medium containing 50 µM fenofibrate was added to the dishes. After 14 days, cells were fixed and stained with crystal violet. Relative density of clones was quantified using ImageJ (Version 1.44p). Experiments were performed in duplicate.

### 3.10. Statistical analysis

Data are presented as mean ± SD. Comparison between two experimental groups was performed using the Student’s t-test. *P*-values ≤ 0.05 were considered statistically significant.

## 4. Conclusions

Glioblastoma multiforme is a tumor characterized by rapid proliferation, metastatic spread, and resistance to chemo- and radiotherapy. CYR61 appears to be a key regulator of cell migration and proliferation in various types of cancer, including Glioblastoma [9,19].

Among the various microRNAs that are predicted to target CYR61, miR-155 has been shown to inhibit the 3’UTR of CYR61 [24], and we originally selected this microRNA as a positive control in our experiments. Although it has never been validated, miR-136 and miR-634 are also predicted to target the 3’UTR of CYR61, and our results validated this prediction (Figure 1). We also reasoned that up-regulation of these microRNAs by fenofibrate could be responsible for the decrease in CYR61 observed following fenofibrate treatment (Figure 5A and B). Although our results did not reveal any significant up-regulation in the expression of these miRNAs by fenofibrate, CYR61-targeting microRNAs may have still the potential to enhance the antitumor activity of fenofibrate or other anti-cancer agents, and we decided to investigate their biological effect on glioblastoma cells. In addition, miR-136 and miR-634 have both been shown to impair the growth of tumor cells [25,26]. In line with those findings, we also found that both miRNAs have an anti-proliferative effect when expressed in LN229 glioblastoma cells (Figure 2A). Although miR-155 did not influence the rate of cell growth, this microRNA effectively impaired the cell migration (Figure 2B and C). This result is also in agreement with previous reports where miR-155 has been shown to hinder the migration of multiple cell types [24,43,44]. Interestingly, the addition of CYR61 to the migration assay did not reverse the impairment of migration induced by miR-155 (Figure 2C), suggesting that this microRNA may affect other targets/pathways necessary for cell migration. For instance, miR-155 has been found to inhibit migration of HTR-8/SVneo cells and cardiomyocyte progenitor cells by targeting Cyclin D1 and MMP-16, respectively [44,45].

In the attempt to characterize the molecular mechanisms implicated in the differential effects of the three miRNAs on proliferation and migration of LN229 cells, we investigated the phosphorylation status of three key factors typically involved in those cellular functions: AKT, ERK1/2 and p70S6K. Our results (Figure 3) were disappointing, as the activation of all three molecules was actually potentiated by miRNA expression. Further analysis indicated that, at least for the miR-634-dependent activation of p70S6K the phosphorylation state and amount of TSC2 in LN229 cells overexpressing this miRNA was reduced (Figure 4). This result demonstrates that the miR-634-induced up-regulation of mTOR activity is at least partially dependent on the phosphorylation and degradation of TSC2 by ERK1/2. We did not observe this degree of TSC2 down-regulation in cells over-expressing miR-136 and miR-155. As miR-136 targets PTEN [46], an upstream negative regulator of mTOR, this could be a possible mechanism to explain the activation of mTOR in cells expressing this miRNA. However, we may have to exclude this possibility since we did not observe appreciable increase in AKT phosphorylation (Figure 3C). Reduction of IRS1 expression could also explain impairment in the proliferative response in the presence of mTOR activation; however, we did not observe changes in levels of this protein in our experimental setting (data not shown).

Fenofibrate is an FDA approved PPARα agonist that has previously been shown to block the proliferation and migration of glioblastoma [3,39]. In the present study we investigated the effect of fenofibrate on gene expression in the glioblastoma cell line, LN229. Our gene array results revealed that the expression of CYR61 was down-regulated in response to treatment with fenofibrate (Figure 5). As CYR61 has been shown to increase the proliferation and migratory capacity of tumor cells, this result suggests that the activity of fenofibrate against tumors may be partially mediated by down-regulating this protein. In the attempt to characterize the mechanisms involved in the fenofibrate-mediated down-regulation of CYR61, we investigated the possible role of our selected microRNAs. However, no changes in the expression of the three miRNAs were observed in fenofibrate treated cells. Interestingly, as the transcription factor FoxO3A has been shown to down-regulate the expression of CYR61 [47] and fenofibrate increases the activity of FoxO3A [39], this may represent a mechanism by which this inhibitor affects levels of CYR61. Regardless of the mechanism for down-regulation, as both miRNAs and fenofibrate target CYR61, we reasoned that over-expression of miRNAs could sensitize cells to fenofibrate treatment.

Fenofibrate has previously been shown to block the clonogenic growth of medulloblastoma and glioblastoma cells [38,39]. We therefore decided to examine the effect of microRNA-induced mTOR activation on clonogenic growth inhibition by fenofibrate. The effect of fenofibrate on the clonogenic growth of cells expressing miR-136 or miR-155 was not statistically different from that of cells expressing empty vector. However, we did find that expression of miR-634 in LN229 cells is partially protective against the anti-clonogenic effects of fenofibrate (Figure 6B). This result is not entirely surprising as previous studies have found a connection between mTOR activation and resistance to chemotherapy. A report by Bostner *et al.* found that increased expression of phospho-mTOR in primary breast tumor samples correlated with a reduced response to tamoxifen [48]. In another study, treatment with rapamycin reversed the doxorubicin resistant phenotype of PTEN-negative prostate cancer cells [49]. In line with these findings, Wendel *et al.* showed that rapamycin blocks doxorubicin chemoresistance in an *in vivo* murine lymphoma model [50]. In addition to mTOR activation, the miR-634-induced up-regulation of ERK1/2 activity could also promote a partial chemoresistant phenotype in tumor cells. For instance, Yoon *et al.* found that chemoresistant Intrahepatic cholangiocarcinoma cells showed increased activation of ERK1/2, along with increased AKT activation [51]. Another recent report demonstrated that inhibition of clusterin-dependent ERK1/2 activation sensitized pancreatic cancer cells to gemcitabine treatment [52].

In conclusion, our studies demonstrated that overexpression of CYR61-targeting microRNAs impairs the growth (miR-136 and miR-634) and the migration (miR-155) of Glioblastoma cells. Expression of these microRNAs was accompanied by activation of mTOR pathway signaling, which was dependent upon the activity of AKT, ERK1/2, and mTORC1. In clonogenic growth assays miR-634-expressing cells, but not miR-136 or miR-155, were partially desensitized to the inhibitory effects of fenofibrate. Further investigation will be required to better understand the role of miR-634 and subsequent mTOR activation in the resistance of Glioblastoma to fenofibrate and to identify other signaling molecules critical to this process. Finally, in addition to providing valuable information on the differential effects of microRNAs-136, -155, and -634 on cell growth and migration, our studies have generated further mechanistic insights into the activity of fenofibrate against Glioblastoma.
